# Long-Term Outcomes Associated with NAFLD, ASCVD, and All-Cause Mortality of Patients with Metabolic Syndrome

**DOI:** 10.3390/jcm11154627

**Published:** 2022-08-08

**Authors:** Suchanart Jitrukthai, Chayanis Kositamongkol, Punyisa Boonchai, Euarat Mepramoon, Pinyapat Ariyakunaphan, Pongpol Nimitpunya, Weerachai Srivanichakorn, Thanet Chaisathaphol, Chaiwat Washirasaksiri, Chonticha Auesomwang, Tullaya Sitasuwan, Rungsima Tinmanee, Naruemit Sayabovorn, Phunchai Charatcharoenwitthaya, Pochamana Phisalprapa

**Affiliations:** 1 Division of Ambulatory Medicine, Department of Medicine, Faculty of Medicine Siriraj Hospital, Mahidol University, Bangkok 10700, Thailand; 2 Division of Gastroenterology, Department of Medicine, Faculty of Medicine Siriraj Hospital, Mahidol University, Bangkok 10700, Thailand

**Keywords:** Asian, atherosclerotic cardiovascular diseases, long-term outcomes, metabolic syndrome, mortality, nonalcoholic fatty liver disease, transient elastography, ultrasonography

## Abstract

Metabolic syndrome (MetS) patients are at higher risk for nonalcoholic fatty liver disease (NAFLD), atherosclerotic cardiovascular diseases (ASCVD), and death. Given a lack of longitudinal data on patients with MetS in Southeast Asia, this study investigated the incidence of NAFLD and ASCVD and the all-cause mortality rate during a 10-year follow-up of Thai patients with MetS. Retrospective data were collected on 496 MetS patients with ultrasonography or transient elastography results. The patients had been followed up continuously by a university hospital between October 2011 and November 2021, and their mean age was 61.0 ± 10.9 years. Patients with secondary causes of hepatic steatosis were excluded. Cox proportional hazards regression models with time-varying covariates were adopted. During the 10-year follow-up, 17 patients (11.2%) developed NAFLD, and 27 (6.4%) developed ASCVD. The NAFLD and ASCVD incidence rates were 21.7 and 10.9 events per 1000 person years, respectively. The mortality rate was 14.2 deaths per 1000 person years. The prevalence of hypertension, dyslipidemia, ASCVD, NAFLD, advanced fibrosis, and cirrhosis at baseline was significantly higher in the nonsurvival group. The NAFLD incidence and mortality rate of patients with MetS were lower than those in previous studies. Intensive, holistic, and continuous care should be considered for better outcomes.

## 1. Introduction

The global prevalence of nonalcoholic fatty liver disease (NAFLD) diagnosed by an imaging test has been estimated to be 25% in the general population. In Asia, its prevalence was estimated to be approximately 27%. The prevalence of NAFLD is increasing worldwide and is expected to be 33.5% in 2030, affecting 100.9 million people [1]. Its prevalence is significantly higher in specific groups of patients: those with metabolic syndrome (MetS), obesity, type 2 diabetes, hypertension, and dyslipidemia [2,3,4,5,6,7,8,9,10]. The prevalence of NAFLD was estimated to be 63% in patients with MetS and 57.8% in patients with type 2 diabetes [11]. NAFLD has become one of the most common causes of chronic liver disease and causes substantial global clinical and economic burdens [2,12,13].

NAFLD is characterized by excessive hepatic fat accumulation and is defined by the presence of steatosis in more than 5% of hepatocytes. NAFLD can be divided pathologically into nonalcoholic fatty liver (NAFL) and nonalcoholic steatohepatitis (NASH); the latter is a more severe disease state [14,15]. Many studies have demonstrated that NAFLD and MetS have a bidirectional association. MetS increased the risk of developing NAFLD, and the characteristics of MetS were highly found in patients with NAFLD [2,16]. NAFLD also had many consequences. It increased the risks of hepatic-related diseases (such as cirrhosis and hepatocellular carcinoma) and extrahepatic consequences (such as atherosclerotic cardiovascular diseases (ASCVD) and chronic kidney disease) [17,18]. Recently, colonic diverticulosis also found to be associated with NAFLD and MetS [19]. Patients with NAFLD also had an almost doubled mortality risk [17,18,20]. The mortality rate of patients with biopsy-proven NAFLD was 16.9 deaths per 1000 patient years, whereas the rate in the general population was 11.7 deaths per 1000 person years. An adjusted hazard ratio of 1.93 was reported for the overall mortality of NAFLD compared with the general population in Europe [19].

NAFLD is associated with risks of both fatal and nonfatal ASCVD events. According to previous evidence, patients with more severe NAFLD were more likely to develop fatal and nonfatal ASCVD events [14]. Additionally, MetS on its own is associated with increased ASCVD risk, ASCVD-related death, and all-cause mortality [21,22,23]. Although many studies reported that NAFLD was associated with MetS, there are still limited longitudinal data on NAFLD incidence in patients with MetS in the Asian population. This study focused on the incidence of NAFLD in patients with existing MetS during a 10-year follow-up period. Additionally, the study examined the incidence of ASCVD and all-cause mortality in Thai patients with MetS since there are limited related data for the Thai population.

## 2. Materials and Methods

### 2.1. Study Design and Ethics Approval

This retrospective longitudinal cohort study enrolled patients treated by the outpatient department or the continuity-of-care clinic of Siriraj Hospital, Thailand, between October 2011 and December 2013. Siriraj is a 2221-bed tertiary care university hospital [24]. The cohort was followed up until November 2021. The study protocol was approved by the Siriraj Institutional Review Board (approval number: Si 540/2011).

### 2.2. Patient Selection

All consecutive patients who visited the hospital during the recruitment period and met the eligibility criteria were included in the cohort. The inclusion criteria were as listed below.

- A Thai adult patient (age ≥ 18 years).
- Diagnosed with MetS per the guidelines of the National Cholesterol Education Program Adult Treatment Panel III (NCEP ATP III) 2005 [25,26] and the American Heart Association/National Heart Lung and Blood Institute (AHA/NHLBI) 2005 [27]. The diagnostic criteria are described elsewhere [25,26,27]. In this study, the waist circumference cutoffs used to diagnose MetS were those for the Asian population: ≥ 80 cm for women and ≥90 cm for men [26].
- Underwent abdominal ultrasonography or transient elastography at least once at baseline.

Patients were excluded if they had any secondary cause of hepatic steatosis such as a previous or current excessive alcohol intake (exceeding 30 g/day for men and 20 g/day for women), viral hepatitis, chronic liver disease, or drug-induced hepatitis [14,28,29]. A review of data on patients’ diagnoses, laboratory results, and drug prescriptions in the hospital’s electronic database was performed to identify the patients to be excluded.

### 2.3. Data Collection

Internal medicine residents collected the data of the included patients through a retrospective review process via the electronic database. The collected data comprised demographic profiles (age, sex, weight, body mass index (BMI), anthropomorphic data, and smoking status) and comorbidities. All abdominal ultrasonography and transient elastography results recorded at the enrollments and during the patients’ 10-year follow-ups were also collected. Office blood pressure and blood chemistry parameters (fasting blood sugar (FBS), hemoglobin A1c (HbA1c), lipid profiles, blood proteins, hepatic enzymes, complete blood count, and kidney profiles) measured at the abdominal ultrasonography or transient elastography dates were reviewed. Data were deemed missing if there were none for the date of an abdominal investigation or within 6 months before or after that date. In such cases, the missing data were imputed using the last observation carried forward method. All clinical and laboratory data were measured using standard techniques. Documented diagnosis dates of ASCVD and date of death were retrieved from the electronic databases of Siriraj Hospital and the Civil Registration Office, respectively.

### 2.4. Definition of Outcomes and Assessments

#### 2.4.1. Nonalcoholic Fatty Liver Disease and Fibrosis

Cases of NAFLD were diagnosed by abdominal ultrasonography or transient elastography (FibroScan; Echosens, Paris, France), performed by a radiologist and a gastroenterologist, respectively. Patients were defined as having NAFLD if they had a bright liver score ≥ 1, a controlled attenuation parameter value > 275 dB/m, or a liver stiffness measurement ≥ 7.0 kPa [30].

The present study diagnosed advanced fibrosis and cirrhosis, both at the time of enrollment and during the follow-up, when the liver stiffness measurement value from transient elastography was ≥ 8.0 kPa and ≥ 10.3 kPa, respectively [30,31].

#### 2.4.2. Atherosclerotic Cardiovascular Diseases

The ASCVD collected in this study comprised coronary heart disease, cerebrovascular disease (transient ischemic attack, ischemic stroke, and hemorrhagic stroke), and peripheral arterial disease. A patient diagnosed with one or more was considered to have ASCVD.

#### 2.4.3. Mortality Status

Since we could not identify the cause of death of the included patients, all-cause mortality was another outcome of interest in this study. Patients’ living statuses and death dates during the follow-up period were retrieved from the electronic database of the Civil Registration Office.

### 2.5. Statistical Analysis

Demographic data were analyzed and reported using descriptive statistics. Frequency and percentage were used to summarize categorical variables. Normally distributed continuous variables are expressed as the mean with standard deviation. Skewed continuous data are reported as the median with an interquartile range.

The baseline characteristics of patients with NAFLD were compared with those without NAFLD. In addition, the differences between the baseline characteristics of living and deceased patients were examined. Independent t-tests were used to compare the normally distributed continuous variables of the two groups. Mann–Whitney U tests were used to compare nonnormally distributed continuous variables. Proportional data of the two groups were compared using Fisher’s exact test.

Survival analyses of failure events (death rate of patients with MetS, NAFLD incidence, and ASCVD incidence in patients at risk) were performed. Kaplan–Meier curves were used to estimate survival probabilities during the follow-up period of the cohort. The incidence rates of the two groups were compared by log-rank test. Cox proportional hazards regression models with time-varying covariates were adopted to determine which parameters were associated with the patients’ failure outcomes. A probability-value (*p*-value) of less than 0.05 was considered statistically significant. All analyses were performed using Stata Statistical Software, release 15.1 (StataCorp LLC, College Station, TX, USA).

## 3. Results

### 3.1. Demographic Data

In all, 509 patients with MetS underwent abdominal ultrasonography or transient elastography during the recruitment period. Of these, a secondary cause of hepatic fat accumulation was identified in 13 patients. The final cohort for analyses was left with 496 patients who complied with the eligibility criteria. Their average age at inclusion was 61.0 ± 10.9 years. The median follow-up was 9.0 years (8.4, 9.5), while the total number of abdominal ultrasonography and transient elastography visits was 772. Among the included patients, 344 (69.4%) were diagnosed with NAFLD at baseline, and 152 (30.6%) were placed in the non-NAFLD group.

Table 1 shows the baseline characteristics of patients with and without NAFLD at inclusion. The age at inclusion, weight, BMI, waist circumference, and hip circumference of patients with NAFLD were significantly higher than those without NAFLD. More than half of the patients in the NAFLD group had type 2 diabetes, with a significantly higher prevalence than in the non-NAFLD group (56.4% versus 34.2%, *p*-value < 0.001). Significantly greater FBS, HbA1c, triglycerides, aspartate aminotransferase (AST), alanine aminotransferase (ALT), gamma-glutamyl transferase, and albumin were found in patients with NAFLD. High-density lipoprotein cholesterol (HDL-C) and creatinine levels were significantly lower in patients with NAFLD than those without NAFLD. Surprisingly, the non-NAFLD group was associated with a higher proportion of patients with existing ASCVD than the NAFLD group (27.6% versus 16.0%, *p*-value = 0.003).

### 3.2. Incidence of NAFLD

**Figure 1:**
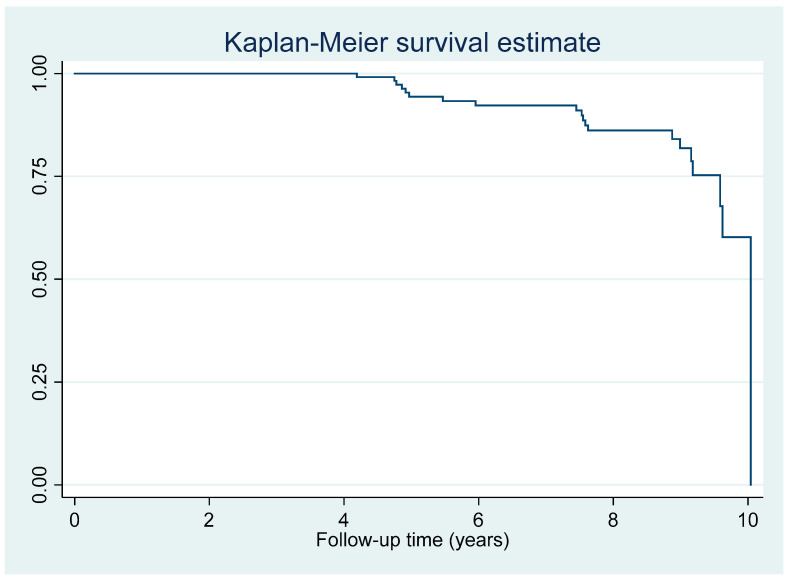
Kaplan–Meier survival curve of nonalcoholic fatty liver disease incidence in patients with metabolic syndrome.

Among 152 patients without NAFLD at baseline, 17 patients developed NAFLD during the follow-up period (incidence proportion = 11.2%). The NAFLD incidence rate in patients with MetS was 21.7 events per 1000 person years. The estimated survival probability during the follow-up period was shown in a Kaplan–Meier curve (Figure 1).

### 3.3. Mortality Rate and Clinical Differences of Patients with Metabolic Syndrome

Among 496 included patients with MetS, 55 patients (11.1%) died during the 10-year follow-up period. The nonsurvival group was older and had a significantly larger waist circumference than those who survived. The prevalence of hypertension, ASCVD, advanced fibrosis, and cirrhosis at baseline were significantly higher in the nonsurvival group. History of smoking was significantly higher in the nonsurvival group than the survival group (21.8% versus 6.8%, *p*-value < 0.001). LSM value at baseline was significantly higher for the nonsurvival group than the survival group (5.9 kPa [5.1, 9.8] versus 4.8 kPa [4.0, 5.9], *p*-value < 0.001). The details are in Table 2.

**Figure 2:**
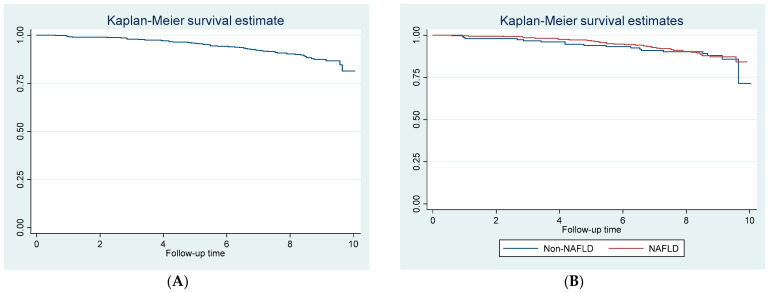
Kaplan–Meier curves of mortality outcome for (**A**) the whole cohort and (**B**) subgroups of patients with and without nonalcoholic fatty liver disease.

Mortality rate of the patients with MetS was 14.2 deaths per 1000 person years. Mortality rates of patients with and without NAFLD did not differ (13.8 and 15.1 events per 1000 person years, respectively, *p*-value = 0.774). Kaplan–Meier curves for the whole cohort and subgroups of patients with and without NAFLD are illustrated in Figure 2.

The Cox proportional hazards regression model with time-varying covariates demonstrated that age, BMI, smoking history, AST/ALT ratio, and globulin were associated with a higher mortality rate, as shown in Table 3.

### 3.4. Incidence of ASCVD

**Figure 3:**
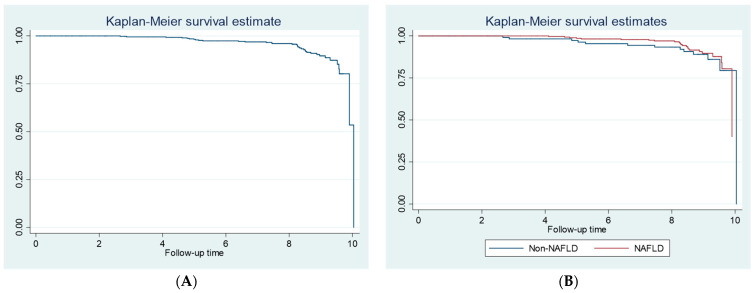
Kaplan–Meier curves of atherosclerotic cardiovascular diseases incidence for (**A**) the whole cohort and (**B**) subgroups of patients with and without nonalcoholic fatty liver disease.

Among 421 patients without ASCVD at baseline, 27 patients developed ASCVD during the follow-up period (incidence proportion = 6.4%). ASCVD incidence rate among patients with MetS was 10.9 events per 1000 person years. ASCVD incidence rates of patients with and without NAFLD were not different (9.7 versus 14.1 events per 1000 person years, respectively, *p*-value = 0.526). Kaplan–Meier curves for the whole cohort and subgroups of patients with and without NAFLD are illustrated in Figure 3.

The Cox proportional hazards regression model with time-varying covariates demonstrated that only HDL-C level was associated with ASCVD incidence rate, as shown in Table 4.

## 4. Discussion

Of our cohort of 496 Thai patients with MetS, 69.4% were diagnosed with NAFLD. This prevalence was slightly higher than that of a meta-analysis conducted by Park et al., who reported a pooled NAFLD prevalence among patients with MetS of 63.2% (95% CI: 59.7, 66.6) [11]. They pooled the prevalence from over 10,000 Korean patients with MetS. Our findings demonstrated that the Thai patients with and without existing NAFLD had noticeably different clinical data. Those with NAFLD tended to have more comorbidities and relatively worse blood biochemistry profiles. Among our patients with NAFLD, the prevalence of obesity was significantly higher than that in the non-NAFLD group (72.1% with a mean BMI of 27.9 ± 4.7 kg/m^2^ in the NAFLD group versus 46.1% with a mean BMI of 25.3 ± 3.9 kg/m^2^ in the non-NAFLD group). These proportions correspond with published data [2,11,32]. There were a number of previously published literatures about mechanisms behind the association among obesity, MetS, and NAFLD. For example, Eslam et al., who introduced the term “metabolic associated fatty liver disease (MAFLD)”, explained that fat deposition sites are associated with metabolic risk as well as liver inflammation and fibrosis. Various subtypes of NAFLD were defined based on metabolic phenotypes including insulin resistance, monounsaturated triacylglycerols, free fatty acids, and ceramides in liver. Some genes were also claimed to play a role in NAFLD development such as *PNPLA3* [33]. Our results also support the current knowledge that greater weight, poorly controlled blood sugar levels, and type 2 diabetes are associated with NAFLD [33]. In our investigation, diabetes among MetS patients with NAFLD was more prevalent than among those without NAFLD (56.4% versus 34.2%, *p*-value < 0.001). The prevalence of type 2 diabetes in a Korean study was 14.2% (95% CI: 11.7, 17.2) compared with patients without NAFLD (5.2%, 95% CI: 4.2, 6.5; *p*-value < 0.001) [11]. However, that reported prevalence was not specific to the MetS subgroup. Our work also found that the mean FBS and HbA1c were significantly higher among individuals with NAFLD (127.3 ± 43.0 mg/dL versus 113.3 ± 34.9 mg/dL, *p*-value < 0.001; and 6.8% ± 1.2% versus 6.3% ± 1.1%, *p*-value < 0.001, respectively), correlating with the prevalence of type 2 diabetes. Furthermore, Younossi et al. demonstrated that obesity was present in 51.0% of individuals with NAFLD, while the pooled overall diabetes prevalence estimated among NAFLD patients was 22.5% (95% CI: 17.9, 27.9) [2]. Another observation we noticed during the data collection process was the absence of a hepatocellular carcinoma incidence among patients with existing NAFLD at baseline during the 10-year follow-up (2685 person years). In contrast, a previous study reported an annual incidence of hepatocellular carcinoma in patients with NAFLD of 0.44 per 1000 person years (95% CI: 0.29, 0.66) [2].

Unfortunately, we did not observe an association between either hypertension or dyslipidemia and NAFLD, but the mean blood pressure of patients with NAFLD was significantly higher than that of patients without NAFLD. The nonsignificant outcomes supported a previous meta-analysis. The mentioned study involved more than 3 million participants, and it reported that the comorbidities of hypertension and dyslipidemia failed to show associations with NAFLD [11]. Additionally, our study found that the triglyceride levels of individuals with NAFLD were significantly higher than those without NAFLD (131 mg/dL [96.5, 180.0] versus 99.5 mg/dL [74.5, 138.5], *p*-value < 0.001). Furthermore, the mean HDL-C level of our NAFLD group was 51.6 ± 12.5 mg/dL, which was significantly lower than that of the non-NAFLD group (57.9 ± 16 mg/dL, *p*-value < 0.001). Ma et al. also found these associations, reporting triglycerides as a risk factor for NAFLD (odds ratio: 1.686, 95% CI: 1.329, 2.139, *p*-value < 0.001) in addition to HDL-C (odds ratio: 0.204, 95% CI: 0.097, 0.429, *p*-value < 0.001) [34]. Among patients with NAFLD in our cohort, the AST, ALT, and gamma-glutamyl transferase values were 26.4 ± 13.7 IU/L, 24 IU/L (17.0, 36.0), and 34.0 IU/L (22.0, 57.0), respectively. These values were significantly higher than those for the non-NAFLD patients. Nevertheless, all values were within normal ranges, corresponding to published data [35].

In contrast with existing evidence [33,36], the non-NAFLD group of our cohort was older and associated with a higher proportion of patients with existing ASCVD than the NAFLD group (27.6% versus 16.0%, *p*-value = 0.003).

In this study, the incidence of NAFLD in patients with MetS was 21.7 events per 1000 person years. This value was much lower than that reported by a meta-analysis of Asian populations. Its pooled annual NAFLD incidence rate was 50.9 cases per 1000 person years (95% CI: 44.8, 57.4) [37]. Most primary studies (16 of 18) in the meta-analysis [37] examined the NAFLD incidence in Chinese and Korean populations. Another investigation reported that the NAFLD incidence among patients with MetS was as high as 77.7 events per 1000 person years (95% CI: 63.9, 91.4) [11]. The methods of diagnosis, the studied population characteristics (such as age, sex, obesity, comorbidities, and ethnicity), and the medical management given to patients might alter the incidence rates [33,37]. We did not explore the association between time-varying factors and NAFLD incidence, as the number of patients who developed the event was too small to determine accurate outcomes.

Evidence has shown a potential association between NAFLD and cardiovascular sequelae [33,36]. Surprisingly, we found that the proportion of patients with ASCVD was smaller in the NAFLD group and that being diagnosed with NAFLD was not associated with ASCVD incidence. The latest Mendelian randomization study aimed to explore the causal relationship of NAFLD with cardiovascular disease and did not reveal outcomes supporting current knowledge. The Mendelian study found no association between NAFLD and coronary artery disease, heart failure, or stroke. Its results supported only the association of NAFLD and arterial-stiffness index [38]. Moreover, a matched cohort study on 18 million European adults suggested that the diagnosis of NAFLD in routine practice might not be associated with acute myocardial infarction and stroke after adjusting for cardiovascular-related confounders. Only HDL-C level was associated with ASCVD incidence in our study; lower HDL-C was associated with ASCVD incidence.

We found that the all-cause mortality rate among patients with MetS, either with or without NAFLD, was 14.2 deaths per 1000 person years. This rate is lower than that found by another investigation of over a thousand American adults with MetS [39]. That study reported age- and sex-adjusted all-cause mortality rates of 17.1 deaths per 1000 person years in MetS without diabetes and 21.1 deaths in MetS with diabetes during a 13.3-year follow-up [39]. Conversely, the mortality rate among patients with NAFLD reported in the most recent meta-analysis was as low as 2.6 deaths per 1000 person years (95% CI: 1.1, 4.8). The meta-analysis also showed that there was no difference in all-cause mortality rates among those being diagnosed with or without NAFLD (2.6 [95% CI: 1.1, 4.8] versus 2.0 deaths per 1000 person years [95% CI: 1.0, 3.3], respectively, *p*-value = 0.42) [11]. Another meta-analysis by Younossi et al. in 2016 [2] reported a nonsignificant pooled incidence rate ratio between the two groups of 1.05 (95% CI: 0.70, 1.56). These data were pooled from five primary studies with a follow-up duration of 13.4 years. Our finding was similar in that we also did not identify a difference in the all-cause mortality rates of these two groups (*p*-value = 0.774). Despite the comparable follow-up durations of previous studies [2,11], inconsistent mortality rates and interpretations were evident. Additionally, our findings demonstrated that age, BMI, smoking history, AST/ALT ratio, and globulin were associated with the mortality of patients with MetS.

The management of the studied patients impacted the mortality outcome and other clinical endpoints. Even though there are international standardized treatment guidelines that healthcare practitioners should follow, minor to substantial variations might be found in routine practice in different healthcare settings. Additionally, many patient-associated factors, such as age and comorbidities, might influence the results.

The source of a proportion of our included patients was the continuity-of-care clinic of a university hospital. Internal medicine specialists at the clinic provided continuous, intensive, and holistic care to every patient. Patients were routinely followed up via 3- to 6-month visits. This approach certainly had an impact on our reported outcomes. We suggest that favorable clinical outcomes are dependent on the intensity of care and patient adherence and compliance.

This study was composed of a relatively large sample size of patients with MetS compared with other studies conducted in Southeast Asia. The follow-up duration was also long enough to investigate the long-term clinical outcomes, including NAFLD and ASCVD incidence rates and mortality of patients with MetS. Moreover, our study used time-varying covariate analysis instead of time-constant covariate analysis. This approach might produce more sensible outcomes. We believe the present study is an excellent example of the continuity-of-care strategy in routine practice. Thus, our findings for the incidence rates of NAFLD and ASCVD, as well as mortality rates, were relatively lower than most previously published studies.

Despite the strengths, our study has several limitations. First, NAFLD and cirrhosis were diagnosed without confirmation from liver biopsy results. Although a liver biopsy is the gold standard for NAFLD diagnosis, the procedure is invasive, frequently associated with distress, and improper for asymptomatic patients. For a large-scale study, such as the current investigation, qualified noninvasive tests are preferred because they are more practicable than a biopsy. Abdominal ultrasonography proved to be a reliable and accurate detection of moderate to severe fatty liver, with an area under the receiving operating characteristics curve of 0.93 [40]. Two studies that developed scores for predicting NAFLD with fibrosis diagnosed by liver biopsy and transient elastography had quite similar parameters [41,42]. It can, therefore, be inferred that transient elastography is an acceptable tool for diagnosing NAFLD. In this present study, the result of ultrasonography was interpreted by only one radiologist. However, the use of transient elastography, which is considered a method with good reproducibility, should increase the sensitivity of detecting NAFLD and liver fibrosis. Patients who had either one of the two tests being positive were defined as having outcomes. Additionally, because we chose noninvasive methods to diagnose NAFLD, NASH, a more specific and severe form of the hepatic condition, could not be identified in our cohort. Second, this study was performed at a single center and a specific clinic within a university hospital. Consequently, the findings might not be generalizable. Moreover, factors related to lifestyle of this present population might be different from another cohort or even another country. The factors such as sleep, rest, and stress were not included in our data collection design. Variation in these lifestyle-related parameters might impact the outcomes [43,44]. The knowledge gap about whether diverse lifestyles and living habits of the population in different countries are associated with MetS and NAFLD is an interesting issue to be explore in a future study. Third, our study had some drawbacks due to its retrospective design. We were unable to control various factors which might have impacts on the outcomes. Additionally, this study did not investigate many factors and confounders claimed by other investigators to be associated with NAFLD incidence and NAFLD-related outcomes. For example, insulin resistance index, hyaluronic acid, cytokeratin-18 fragments, and apolipoprotein A1 were reported as predictors of NASH [31,45,46,47,48]. However, the related laboratory investigations are not practicable, as they are expensive and not commonly available in general practice. In addition, the causal relationships between these factors and NAFLD are not yet completely understood. Fourth, the risk of infection among patients with existing NAFLD is another interesting outcome. There was evidence that reported the higher infection rate in patients with NAFLD, particularly in those with advanced fibrosis, compared to those without NAFLD [49,50]. Unfortunately, we had limited accessibility to the parameters related to infectious events of our participants.

## 5. Conclusions

In our study, the incidence of NAFLD and the mortality rate in patients with MetS were lower than those in previous studies. This discrepancy may be due to intensive, holistic, continuous care in the continuity-of-care clinic at Siriraj Hospital, which should be applied in standard practice for improved outcomes. Among the nonsurvival group, the baseline prevalence of hypertension, ASCVD, advanced fibrosis, and cirrhosis was significantly higher than that in the survival group. The ASCVD incidence and mortality rates of patients with and without NAFLD did not differ. Age, BMI, smoking history, AST/ALT ratio, and globulin level were associated with mortality. HDL-C was the only factor associated with ASCVD incidence.

## Figures and Tables

**Table 1:** Baseline clinical characteristics of patients with and without nonalcoholic fatty liver disease.

Characteristic	Non-NAFLD (n = 152)	NAFLD (n = 344)	*p*-Value
	Mean ± SD	Mean ± SD	
Age (years)	63.7 ± 10.0	59.8 ± 11.1	<0.001
Sex: male (n, %)	73 (48.0)	154 (44.8)	0.558
Weight (kg)	65.0 ± 11.9	72.1 ± 14.0	<0.001
BMI (kg/m^2^)	25.3 ± 3.9	27.9 ± 4.7	<0.001
Obesity * (n, %)	70 (46.1)	248 (72.1)	<0.001
Waist circumference (cm)	88.6 ± 10.5	95.0 ± 10.3	<0.001
Hip circumference (cm)	97.9 ± 8.5	101.2 ± 8.5	<0.001
Smoking (n, %)	18 (11.8)	24 (7.0)	0.080
Hypertension (n, %)	134 (88.2)	314 (91.3)	0.323
Type 2 diabetes (n, %)	52 (34.2)	194 (56.4)	<0.001
Dyslipidemia (n, %)	144 (94.7)	326 (94.8)	1.000
ASCVD (n, %)	42 (27.6)	55 (16.0)	0.003
SBP (mmHg)	130.2 ± 14.1	133.1 ± 14.6	0.044
DBP (mmHg)	76.8 ± 11.0	80.4 ± 11.1	0.001
FBS (mg/dL)	113.3 ± 34.9	127.3 ± 43.0	<0.001
HbA1c (%)	6.3 ± 1.1	6.8 ± 1.2	<0.001
Total cholesterol (mg/dL)	178.2 ± 37.0	180.2 ± 35.3	0.561
Triglycerides (mg/dL), median (IQR)	99.5 (74.5, 138.5)	131 (96.5, 180.0)	<0.001
HDL-C (mg/dL)	57.9 ± 16.9	51.6 ± 12.5	<0.001
LDL-C (mg/dL)	97.9 ± 31.6	98.5 ± 31.7	0.859
TB (mg/dL)	0.53 ± 0.23	0.53 ± 0.24	0.949
DB (mg/dL)	0.20 ± 0.09	0.21 ± 0.10	0.376
AST (IU/L)	21.1 ± 5.2	26.4 ± 13.7	<0.001
ALT (IU/L), median (IQR)	17 (12.5, 21.0)	24 (17.0, 36.0)	<0.001
AST/ALT ratio	1.28 ± 0.41	1.00 ± 0.36	<0.001
GGT (IU/L), median (IQR)	23.0 (18.0, 31.0)	34.0 (22.0, 57.0)	<0.001
Globulin (g/dL)	3.3 ± 0.4	3.3 ± 0.4	0.639
Albumin (g/dL)	4.35 ± 0.28	4.44 ± 0.29	<0.001
Creatinine (mg/dL)	1.01 ± 0.40	0.95 ± 0.31	0.042
LSM (kPa), median (IQR)	4.5 (3.6, 5.2)	5.3 (4.3, 6.5)	<0.001
Advanced fibrosis (n, %)	0 (0.0)	35 (13.6)	<0.001
Cirrhosis (n, %)	0 (0.0)	27 (10.5)	<0.001

* Obesity is defined by BMI ≥ 25 kg/m^2^. ALT, alanine aminotransferase; ASCVD, atherosclerotic cardiovascular diseases; AST, aspartate aminotransferase; BMI, body mass index; DB, direct bilirubin; DBP, diastolic blood pressure, FBS, fasting blood sugar; GGT, gamma-glutamyl transferase; HbA1c, hemoglobin A1c; HDL-C, high-density lipoprotein cholesterol; LDL-C, low-density lipoprotein cholesterol; LSM, liver stiffness measurement; NAFLD, nonalcoholic fatty liver disease; SBP, systolic blood pressure; TB, total bilirubin; TG, triglycerides.

**Table 2:** Baseline clinical characteristics of survival and nonsurvival patients.

Characteristic	Alive (n = 441)	Deceased (n = 55)	*p*-Value
	mean ± SD	mean ± SD	
Age (years)	59.9 ± 10.6	69.5 ± 9.9	<0.001
Sex: male (n, %)	197 (44.4)	30 (54.6)	0.197
Weight (kg)	70.0 ± 13.4	69.9 ± 16.4	0.978
BMI (kg/m^2^)	27.1 ± 4.5	27.1 ± 5.3	0.968
Obesity * (n, %)	284 (64.4)	34 (61.8)	0.766
Waist circumference (cm)	92.7 ± 10.4	95.9 ± 12.7	0.038
Hip circumference (cm)	100.1 ± 8.5	100.6 ± 10.0	0.693
Smoking (n, %)	30 (6.8)	12 (21.8)	<0.001
Hypertension (n, %)	349 (89.3)	54 (98.2)	0.030
Type 2 diabetes (n, %)	231 (48.3)	33 (60)	0.116
Dyslipidemia (n, %)	415 (94.1)	55 (100)	0.099
ASCVD (n, %)	79 (17.9)	18 (32.7)	0.018
SBP (mmHg)	131.9 ± 14.0	134.6 ± 17.9	0.191
DBP (mmHg)	79.6 ± 11.2	76.9 ± 11.1	0.088
FBS (mg/dL)	122.6 ± 41.8	126.6 ± 36.4	0.498
HbA1c (%)	6.6 ± 1.2	6.9 ± 1.2	0.137
Total cholesterol (mg/dL)	180.7 ± 35.3	170.9 ± 38.7	0.055
Triglycerides (mg/dL), median (IQR)	119.0 (87.0, 169.0)	133.0 (94.0, 172.0)	0.366
HDL-C (mg/dL)	53.9 ± 14.2	50.7 ± 15.1	0.122
LDL-C (mg/dL)	99.1 ± 31.3	92.0 ± 34.3	0.116
TB (mg/dL)	0.53 ± 0.23	0.57 ± 0.28	0.250
DB (mg/dL)	0.21 ± 0.09	0.22 ± 0.12	0.289
AST (IU/L)	24.5 ± 11.6	26.8 ± 15.1	0.184
ALT (IU/L), median (IQR)	21.0 (16.0, 31.0)	18.0 (12.0, 30.0)	0.017
AST/ALT ratio	1.1 ± 0.4	1.3 ± 0.5	<0.001
GGT (IU/L), median (IQR)	29.0 (20.0, 48.0)	29 (20.0, 47.0)	0.986
Globulin (g/dL)	3.3 ± 0.4	3.4 ± 0.4	0.054
Albumin (g/dL)	4.4 ± 0.3	4.3 ± 0.3	<0.001
Creatinine (mg/dL)	0.95 ± 0.32	1.14 ± 0.42	<0.001
NAFLD (n, %)	307 (69.6)	37 (67.3)	0.757
LSM (kPa), median (IQR)	4.8 (4.0, 5.9)	5.9 (5.1, 9.8)	<0.001
Advanced fibrosis (n, %)	25 (7.6)	10 (29.4)	<0.001
Cirrhosis (n, %)	19 (5.7)	8 (23.5)	0.002

* Obesity is defined as BMI ≥ 25 kg/m^2^. ALT, alanine aminotransferase; ASCVD, atherosclerotic cardiovascular diseases; AST, aspartate aminotransferase; BMI, body mass index; DB, direct bilirubin; DBP, diastolic blood pressure, FBS, fasting blood sugar; GGT, gamma-glutamyl transferase; HbA1c, hemoglobin A1c; HDL-C, high-density lipoprotein cholesterol; LDL-C, low-density lipoprotein cholesterol; LSM, liver stiffness measurement; NAFLD, nonalcoholic fatty liver disease; SBP, systolic blood pressure; TB, total bilirubin; TG, triglycerides.

**Table 3:** Cox proportional hazards regression model with time-varying covariates for mortality outcome.

Covariates	Adjusted Hazard Ratios	95% CI	*p*-Value
Age	1.06	1.03, 1.10	<0.001
Sex: male	1.87	0.86, 4.09	0.116
BMI	1.08	1.00, 1.16	0.049
Smoking	6.62	2.70, 16.26	0.000
NAFLD at baseline	2.01	0.93, 4.33	0.075
ASCVD	1.44	0.68, 3.04	0.339
Type 2 diabetes	1.08	0.57, 2.06	0.812
AST/ALT ratio	2.40	1.07, 5.37	0.034
Globulin	3.45	1.58, 7.55	0.002
Triglycerides	1.00	0.99, 1.00	0.336
HDL-C	1.02	1.00, 1.05	0.095
Creatinine	1.59	0.73, 3.48	0.244

ALT, alanine aminotransferase; ASCVD, atherosclerotic cardiovascular diseases; AST, aspartate aminotransferase; BMI, body mass index; HDL-C, high-density lipoprotein cholesterol; NAFLD, nonalcoholic fatty liver disease.

**Table 4:** Cox proportional hazards regression model with time-varying covariates for atherosclerotic cardiovascular disease outcome.

Covariates	Adjusted Hazard Ratios	95% CI	*p*-Value
Age	1.00	0.96, 1.03	0.892
Sex: male	1.62	0.78, 3.35	0.193
BMI	0.94	0.85, 1.04	0.225
Smoking	1.02	0.23, 4.46	0.982
NAFLD at baseline	0.82	0.38, 1.76	0.606
Type 2 diabetes	0.63	0.31, 1.27	0.197
HDL-C	0.97	0.94, 1.00	0.023

BMI, body mass index; HDL-C, high-density lipoprotein cholesterol; NAFLD, nonalcoholic fatty liver disease.

## Data Availability

The data presented in this study are available on request from the corresponding author. The data are not publicly available due to privacy and ethical reasons.

## Abbreviations

ALT, alanine aminotransferase; AST, aspartate aminotransferase; BMI, body mass index; CI, confidence interval; DLP, dyslipidemia; DM, diabetes mellitus; FBS, fasting blood sugar; HbA1c, hemoglobin A1c; HDL-C, high-density lipoprotein cholesterol; kPa: kilopascals; LDL-C, low-density lipoprotein cholesterol; MetS, metabolic syndrome; NAFLD, nonalcoholic fatty liver disease; TC, total cholesterol.
